# Implications of atmospheric conditions for analysis of surface temperature variability derived from landscape-scale thermography

**DOI:** 10.1007/s00484-016-1234-8

**Published:** 2016-08-25

**Authors:** Albin Hammerle, Fred Meier, Michael Heinl, Angelika Egger, Georg Leitinger

**Affiliations:** 1 University of Innsbruck, Innsbruck, Austria; 2 Departement of Ecology, Technische Universtität Berlin, Berlin, Germany

**Keywords:** Surface temperature, Thermal infrared camera, Atmospheric correction, Digital elevation model, Alpine environment

## Abstract

Thermal infrared (TIR) cameras perfectly bridge the gap between (i) on-site measurements of land surface temperature (LST) providing high temporal resolution at the cost of low spatial coverage and (ii) remotely sensed data from satellites that provide high spatial coverage at relatively low spatio-temporal resolution. While LST data from satellite (LST_sat_) and airborne platforms are routinely corrected for atmospheric effects, such corrections are barely applied for LST from ground-based TIR imagery (using TIR cameras; LST_cam_). We show the consequences of neglecting atmospheric effects on LST_cam_ of different vegetated surfaces at landscape scale. We compare LST measured from different platforms, focusing on the comparison of LST data from on-site radiometry (LST_osr_) and LST_cam_ using a commercially available TIR camera in the region of Bozen/Bolzano (Italy). Given a digital elevation model and measured vertical air temperature profiles, we developed a multiple linear regression model to correct LST_cam_ data for atmospheric influences. We could show the distinct effect of atmospheric conditions and related radiative processes along the measurement path on LST_cam_, proving the necessity to correct LST_cam_ data on landscape scale, despite their relatively low measurement distances compared to remotely sensed data. Corrected LST_cam_ data revealed the dampening effect of the atmosphere, especially at high temperature differences between the atmosphere and the vegetated surface. Not correcting for these effects leads to erroneous LST estimates, in particular to an underestimation of the heterogeneity in LST, both in time and space. In the most pronounced case, we found a temperature range extension of almost 10 K.

## Introduction

Land surface temperature (LST) is a key variable for numerous environmental functions. It represents the combined result of all energy exchange processes between the atmosphere and the land surface. Thus, LST has become a basic requirement for model validation or model constraining in surface energy and water budget modelling on various scales (Kalma et al. 2008; Kustas and Anderson 2009; and references therein). It serves as a metric for soil moisture and vegetation condition in eco/hydrological modelling and environmental monitoring (Czajkowski et al. 2000; Kustas and Anderson 2009) and has been used in the area of thermal anomalies and high-temperature events detection (Sobrino et al. 2009; Teuling et al. 2010). Further, LST data is widely used in urban climate studies to quantify the surface urban heat island and to explore its relationship with urban surface properties and air temperature variability as well as for surface-atmosphere exchange processes in urban environments (Voogt and Oke 2003; Weng 2009).

LST can be retrieved from various platforms and instruments, depending on the application requirements regarding spatial and temporal resolution. Remote sensing platforms provide data with global coverage. They can routinely either provide LST at a coarse spatial resolution at relatively high overpass frequencies (e.g., Terra-MODIS, Aqua-MODIS, NOAA-AVHRR) or provide less frequent but moderate resolution LST data (e.g., Terra-ASTER, Landsat). Recent developments in the thermal remote sensing system even show a trend towards coarser spatial resolutions (e.g., Sentinel mission). Airborne systems on the other hand can provide relatively high temporal as well as high spatial resolution LST information on a regional scale, with the drawback of high costs. Infrared radiometers mounted on site provide LST at any temporal resolution integrated over a given field of view on the expense of spatial coverage.

Thermal infrared (TIR) cameras have been continuously refined since their broad commercial launch in the early 1990s and have found wide application since the 2000s due to lower costs for uncooled focal plane sensor arrays and their improved spatial and thermal resolution (Schuster and Kolobrodov 2004). The high spatial and temporal resolution, the operational simplicity, and increasing data storage capabilities led to an increasing popularity of this system in many ecological research areas (e.g., Hristov et al. 2008; Katra et al. 2007; McCafferty 2007).

While thermal remote sensing has already been widely applied in landscape ecology (Quattrochi and Luvall 1999 and references therein), the demand for high-resolution data (both, temporally and spatially) is unabated. Particularly in alpine landscapes that are characterized by high spatial heterogeneity and temporal dynamics (resulting from small-scale variations in slope, aspect, and altitude), highly resolved LST data are needed (Bertoldi et al. 2010; Heinl et al. 2012; Scherrer and Körner 2010; Scherrer et al. 2011).

All thermal remote sensing data, independent of the instrument used, is influenced by atmospheric conditions and radiative processes along the measurement path (Chandrasekhar 1960). Several atmospheric correction approaches have been established depending on sensor characteristics, e.g., the split window technique (SWT) for multi-channel sensors (Becker and Li 1990; Kerr et al. 1992; Price 1984; Sobrino et al. 1991), where “split window” refers to radiance differences observed by each atmospheric window of the respective TIR channel. There are different SWT algorithms depending upon spectral emissivity, water vapor content, view angle, or purely empirical algorithms. Radiative transfer models together with atmospheric profile data of pressure, temperature, and humidity are often used to determine SWT algorithms or to perform atmospheric corrections of TIR data derived from single-channel sensors (Berk et al. 1998; Richter and Schläpfer 2002; Schmugge et al. 1998). While these methods are commonly applied to data derived from satellite (Dash et al. 2002; Prata et al. 1995) or airborne platforms (Jacob et al. 2003; Lagouarde et al. 2000; Lagouarde et al. 2004), such corrections are not routinely applied in ground-based TIR imagery in natural and urban environments at the landscape scale (Heinl et al. 2012; Scherrer and Körner 2010; Scherrer and Körner 2011; Scherrer et al. 2011; Tonolla et al. 2010; Wawrzyniak et al. 2013; Westermann et al. 2011), partially justified by relatively short atmospheric path lengths. Existing methods for ground-based TIR imagery are either simple, i.e., based on the assumption of a homogenous sensor-target distance and constant atmospheric transmission value (Yang and Li 2009), or more complex by using a radiative transfer code, atmospheric data and under consideration of differences in atmospheric path lengths (Meier and Scherer 2012; Meier et al. 2011; Sugawara et al. 2001).

This paper compares LST data measured from different platforms. The main objective is to quantify the differences between LST data from a ground-based TIR imagery (LST_cam_) and LST data from on-site radiometry (LST_osr_). Subsequently, an empirical model, based on a digital elevation model and measured vertical air temperature profiles, was developed. This model corrects LST_cam_ for atmospheric influences.

Furthermore, we discuss the consequences of neglecting atmospheric influences on LST data derived from ground-based TIR imagery at the landscape scale.

## Methods

The basis of the study was the comparison of surface temperatures measured (i) continuously by infrared radiometers mounted above the canopy (on-site radiometry), (ii) frequently by a TIR camera operated at an elevated position within the study region (ground-based TIR imagery), and (iii) by satellite remote sensing (satellite-based TIR imagery).

### Study region and experimental setup

**Fig. 1:**
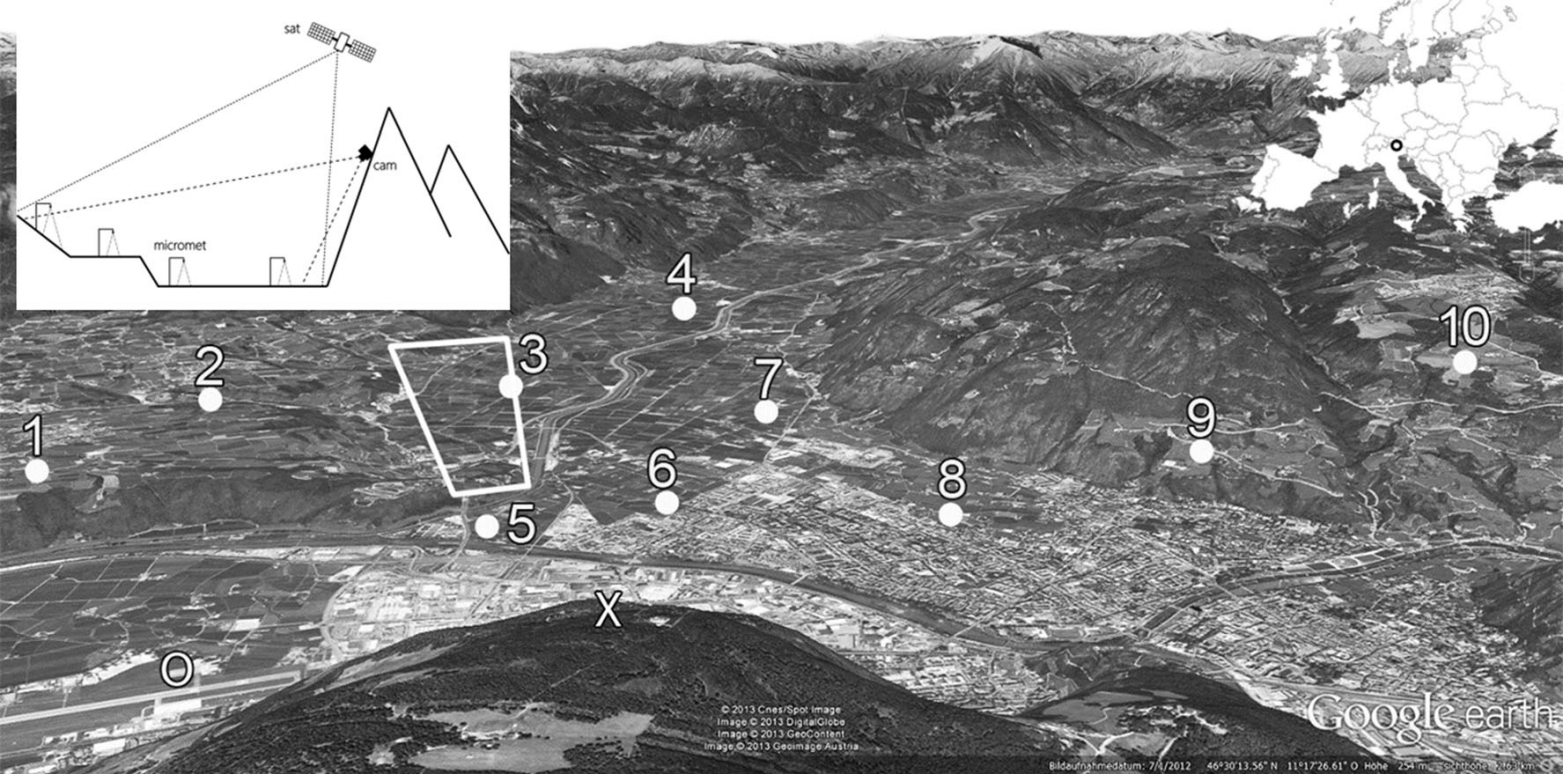
Study area in the basin of Bozen/Bolzano (I). *Numbers* denote locations of on-site measurements and corresponding *numbers* refer to site numbers in Tables 1, 2, and 4. Locations of ground-based TIR imagery and of the microwave radiometer are marked with *X* and *O*, respectively. The *tetragon* within the figure represents the transformed marked section in Fig. 7 and Fig. 8 (*black squares*). *Inset upper left*: schematic overview of the experimental setup. Map data: Google, DigitalGlobe

The study was conducted in the region of Bozen/Bolzano in the northernmost part of Italy (Fig. 1). The city of Bozen/Bolzano is located in a basin at the transition of the central Alps to the southern Alps, surrounded by four mountain ranges. Ten microclimate stations were erected in the vicinity of the city which spanned an elevational range from 239 to 857 m a.s.l. and covered three different land-use types (vineyard, orchard, and grassland).

These three land-use types cover 16, 29, and 4 % of the investigated rural area, respectively (woodland 48 %). Three out of ten microclimate stations were located in vineyards, six in orchards, and one in a managed grassland. While vineyards and orchards are by far the dominating land-use types in this region, grasslands only occurred at higher elevations (Table 1). No site was positioned closer than 20 m to any building.

**Table 1:** Key characteristics of investigated field sites including measures of their positioning relating to the camera position for ground-based TIR imagery (Cam)

Number in Fig. 1	site name	Management	x_GPS	y_GPS	Altitude (m a. s. l.)	Hillslope (°)	Aspect (°)	Situation: cam position-site				
								Atmospheric path length (APL) (m)	Horizontal distance (m)	Altitude difference (m)	Cutting angle (A_C_) (°)	View zenith angle (A_VZ_) (°)
1	Schreckbichl	Vineyard	11.295882	46.460708	474	11.0	63	4624	4584	603	18.3	82.5
2	Girlan	Orchard	11.279210	46.470331	400	3.5	340	5759	5719	678	5.7	83.2
3	Unterrain	Orchard	11.279432	46.488236	240	<3	124	6072	6014	837	8.0	82.1
4	Terlan	Orchard	11.252084	46.511405	243	<3	243	9038	8999	834	5.3	84.7
5	Kaiserau	Orchard	11.313778	46.477665	245	<3	323	3260	3152	833	13.7	75.2
6	Jennerhof	Orchard	11.315806	46.486065	245	<3	240	3457	3355	832	13.3	76.1
7	Moritzing	Orchard	11.299137	46.498861	239	<3	20	5275	5208	838	9.1	80.9
8	Alte Mendl Str	Vineyard	11.329031	46.496685	250	<3	192	3523	3424	827	13.8	76.4
9	Glaninger Weg	Vineyard	11.334097	46.509057	430	21.6	164	4535	4488	647	29.7	81.8
10	Wiesmanhof	Grassland	11.339721	46.524530	857	9.3	149	6049	6045	221	10.8	87.9
X	Kohlern	–	11.353672	46.471006	1077	–	–	–	–	–	–	–

Meteorological measurements included air temperature (*T*_air_) and relative humidity (RH) at 2 m above ground (Hobo Pro v2-U23-002; onset; Bourne, MA, USA), air temperature 1 m above the canopy (PT 100; EMS; Brno, Czech Republic), incoming solar radiation (SR) (S-LIB-M003; onset; Bourne, MA, USA) above the canopy, soil temperature (*T*_soil_) at 0.1 and 0.25 m soil depth (PT 100; EMS; Brno, Czech Republic), and soil water content (SWC) in 0.25 m soil depth (EC-10; Decagon Devices; Pullman, WA, USA). Surface temperatures were derived using an infrared radiometer (SI-111; Apogee Instruments; Logan, UT, USA) mounted 1 m above the canopy. This sensor is sensitive in the electromagnetic spectrum from 8 to 14 μm. Given the half-angle field of view of 22° and the different canopy heights, the visible surface areas ranged from 2 to 8 m^2^. Data were measured every minute and stored as 10 min average values. Land surface temperatures (LST) derived from on-site radiometry are henceforth referred to as LST_osr_.

For ground-based TIR imagery, an elevated site on top of a cliff edge (1077 m a.s.l.) was chosen as camera position (Table 1). Measurements were done using the TIR camera “Jenoptik VarioCAM high resolution” (Infratec; Dresden, Germany), which is sensitive in the electromagnetic spectrum from 7.5 to 14 μm. The camera resolution of 768 × 576 pixels in combination with the standard lens (focal length 25 mm) resulted in pixel sizes ranging from 2.2 to 6.3 m depending on the given atmospheric path length (APL) per site (Table 1).

TIR images were taken on 13 days throughout the summer and autumn season 2012 from an exposed position ca. 840 m above the valley floor. While data were restricted to daytime measurements on some days, we conducted 24-h measurements on others. Measurements were done at least half hourly (higher frequency around sunrise and sunset or at the times of a satellite overpass), resulting in roughly 250 acquisition times where all ten LST_osr_ sites were covered simultaneously. Image processing was done using IRBIS® software (InfraTec; Dresden, Germany). All TIR images were exported as ASCII files and further analyzed using MATLAB (R2013b, The MathWorks, Inc., USA). Despite the mean absolute differences between LST_osr_ and LST_cam_ (0.8 K) being lower than the TIR camera accuracy (±1.5 K), the two systems were intercalibrated in an experimental setup. LST measured by the ground-based TIR imagery are referred to as LST_cam_.

Surface emissivity (*ε*) was considered equal to 1 for both LST_osr_ and LST_cam_ unless specified differently, as pixels of interest were completely covered by vegetation having a high emissivity at all wavelengths.

Satellite-based TIR imagery was derived from ASTER Level 2B03 data products with a spatial resolution (pixel size) of 90 m, acquired on demand for seven dates in 2012 (21 and 28 June 2012; 7 July 2012; 8 and 24 August 2012; 11 and 18 October 2012). The images provide kinetic temperatures at about 11:15 CET and represent the single pixel values at the location of each microclimate station. The standard deviation is calculated over this target pixel and the eight neighboring pixels. Data affected by clouds were not considered for the analyses so that the number of remotely sensed data per site ranges between three and seven observations. LST derived from remote sensing are henceforth referred to as LST_sat_.

A vertical air temperature profile was measured at the airport in Bozen/Bolzano (BZO) using a microwave radiometer (MTP-5HE; ATTEX Ltd., Moscow, Russia) (Fig. 1). This radiometer measured air temperature profiles up to 1000 m above surface (50 m vertical resolution; 10 min time resolution) with a temperature accuracy from ±0.3 K (0–500 m) up to ±0.4 K (>500 m). Radiometer data were provided by “Autonome Provinz Bozen Südtirol/Provincia autonoma die Bolzano Alto Adige” (Landesagentur für Umwelt/Agenzia provinciale per l’ambiente; Labor für physikalische Chemie/Laboratorio di chimica fisica). Average path temperatures (*T*_path_) were calculated for each LST_cam_ measurement as the arithmetic mean over the corresponding temperature profile segment, defined by the site and camera elevation.

### Processing of ground-based TIR imagery

To cover all field sites by ground-based TIR imagery at one time, we had to pan the camera and take five TIR images (scenes). While we always tried to position the camera the same way and choose the same field of view, the different scenes were not perfectly congruent. Thus, we chose one reference thermal image per scene and used the “Computer Vision System Toolbox” of MATLAB (R2013b, The MathWorks, Inc., USA) to align all TIR images of one scene with each other. More precisely, we (i) used the SURF blob detector (detectSURFFeaturs-function; Bay et al. 2008) to identify matching regions in the two TIR images, (ii) estimated the geometric transformation from matching point pairs (estimateGeometricTransform-function; Torr and Zisserman 2000), and (iii) applied the geometric transformation to the TIR image (imwrap-function).

Subsequently transformed TIR images (*n* = 3169) were filtered based on a three-step quality check. (i) All images obviously not matching the corresponding reference TIR image by visual inspection were selected and removed (remaining *n* = 2909; 92 %). (ii) Any TIR image not exceeding a certain *R* ^2^ value (night 0.6; day 0.8), when compared with the reference scene or with less than five matching points found in the SURF blob detector algorithm described above were removed (remaining *n* = 2106; 66 %). (iii) For any averaging interval with multiple LST_cam_ measurements, only the one closest in time to the LST_osr_ measurement was used, further reducing the number of remaining LST_cam_ measurements for the ten sites (remaining *n* = 2011; 63 %). Applying these algorithms and filters resulted in a dataset of TIR image per scene that perfectly matched each other. In order to get the line-of-sight geometry for each TIR image pixel, the procedure described in the following section was applied.

### Derivation of line-of-sight geometry parameters for ground-based TIR imagery

The oblique view of the TIR camera and the topography of the observed landscape produce different line-of-sight (LOS) geometry parameters for each TIR image pixel. The LOS is fully described by APL, by the altitude of the observed surface, and by the view zenith angle (*A*_VZ_) under which the TIR camera observes the surface. The calculation of spatially distributed LOS values for every TIR image pixel is based on the idea that every TIR image pixel has a corresponding 3D geographic coordinate (*x*, *y*, *z*). In order to find these pixel-specific coordinates, the perspective projection of the three-dimensional (3-D) landscape onto the two-dimensional (2-D) TIR image plane was modelled using a DEM of the study region with a spatial resolution of 20 m (Autonomous Province of Bolzano, South Tyrol, Italy). Further, we had to know the geographic coordinates of the TIR camera location and the geographic coordinates of the center pixel of the TIR image (exterior orientation) as well as the size of the 2-D image plane (768 × 576 pixels) and the horizontal and vertical field of view (FOV) of the camera lens (interior orientation). The horizontal FOV is 30° and the vertical FOV is 23°. A detailed description of the perspective projection of the 3-D DEM and calculation of FOV parameters are given in Meier et al. (2011).

### Multiple regression model to correct LST_cam_

Establishing the multiple regression model was done using IBM SPSS Statistics for Windows, Version 21.0 (IBM Corp; Armonk, NY). The LST model was built using ordinary least squares (OLS) regression based on 1839 observations with LST_osr_ as dependent variable and four independent variables (LST_cam_; *T*_path_; difference of LST_cam_ and *T*_path_; APL). (i) All independent variables were tested regarding multi-collinearity, (ii) scatter plots of the dependent vs. each independent variable were analyzed to check for non-linearity, (iii) the significant independent variables were selected by forced entry OLS using ca. 50 % of available data (897 observations; calibration dataset), (iv) a residual analysis was performed for checking OLS assumptions, and finally (v) the LST model was employed to predict the dependent variable for the remaining validation dataset (942 observations; validation dataset).

## Results

Data were collected continuously at the field sites from 1 May 2012 until 31 October 2012. *T*_air_ ranged from −5.2 °C (30 October 2012) to 37.3 °C (20 August 2012), with a mean *T*_air_ of 18.8 °C during that period (30-year average for that period, 19.4 °C (Hydrographisches Amt Bozen/Ufficio idrografico Bolzano)). While the coolest site on average was the Wiesmanhof site, representing the highest-located site (Fig. 1, Table 1), the lowest air temperatures were measured at the Terlan site. The highest air temperature was measured at the Alte Mendel Strasse site, a site located in close proximity to Bozen/Bolzano (Fig. 1, Table 2). LST_osr_ ranged from −5.7 °C (30 October 2012; Terlan) to 49.1 °C (26 July 2012; Wiesmanhof), with an average LST_osr_ of 18.0 °C during the measurement period (Table 2). Average wind speed ranged from 0.8 m s^−1^ (Terlan site) up to 1.5 m s^−1^ (Wiesmanhof), and mean solar radiation (SR) ranged from 196 W m^−2^ (Girlan) to 226 W m^−2^ (Glaninger Weg) among the ten field sites (Table 2).

**Table 2:** Meteorological conditions at the ten field sites throughout the measurement campaign 1 May 2012 until 31 October 2012

Site		*T*_air_ 200 cm (°C)			LST_osr_ (°C)			SR (W m^−2^)	Wind speed (m s^−1^)
Nr.	Name	Min	Max	Mean	Min	Max	Mean	Mean	Mean
1	Schreckbichl	−1.5	35.9	18.6	−3.1	38.6	17.9	206	1.4
2	Girlan	−2.6	35.9	18.2	−2.6	37.8	17.2	196	1.1
3	Unterrain	−3.8	36.9	19.0	−4.4	33.3	17.6	210	1.1
4	Terlan	−5.2	36.5	18.2	−5.7	33.6	17.4	201	0.8
5	Kaiserau	−2.9	36.7	19.5	−3.3	39.1	18.3	202	1.0
6	Jennerhof	−2.4	36.9	19.8	−3.1	36.1	18.7	205	0.8
7	Moritzing	−3.7	37.0	19.2	−3.7	33.8	18.0	209	1.1
8	Alte Mendl Str	−2.3	37.3	20.2	−3.1	40.2	19.4	196	0.9
9	Glaninger Weg	−1.1	36.8	19.3	−2.9	43.0	18.8	226	1.3
10	Wiesmanhof	−3.5	32.3	16.3	−5.0	49.1	16.8	213	1.5

Numbers (nr.) refer to numbers in Fig. 1

*T*_*air*_ air temperature 2 m above ground, *LST*_*osr*_ land surface temperature from on-site radiometry, *SR* shortwave radiation

### LST_sat_ compared to LST_cam_ and LST_osr_

During the measurement campaign, LST_sat_ could be retrieved from seven satellite overpasses. Excluding data with cloud cover, 58 data points could be used from our ten field sites to compare LST_sat_ with LST_osr_ and 32 data points to compare LST_sat_ and LST_cam_. LST_sat_ data are available as kinetic (kin) LST (routinely corrected for atmospheric effects); thus, LST_osr_ and LST_cam_ data had to be recalculated from radiant temperature by applying *ε* = 0.97 (deciduous vegetation and grass; Jensen 2007) and an environmental temperature (*T*_sky_; *K*) that was modelled as a function of vapor pressure (*e*_a_; kPa) and air temperature (*T*_air_; K) following Campbell and Norman (1998) (rearranged):

$$ {T}_{\mathrm{sky}}=\sqrt[4]{1.72\cdot {\left(\frac{e_{\mathrm{a}}}{T_{\mathrm{a}\mathrm{ir}}}\right)}^{\frac{1}{7}}\cdot {\left({T}_{\mathrm{a}\mathrm{ir}}\right)}^4} $$

This intercomparison was done using original LST_cam_ data, not corrected for any atmospheric influences.

**Fig. 2:**
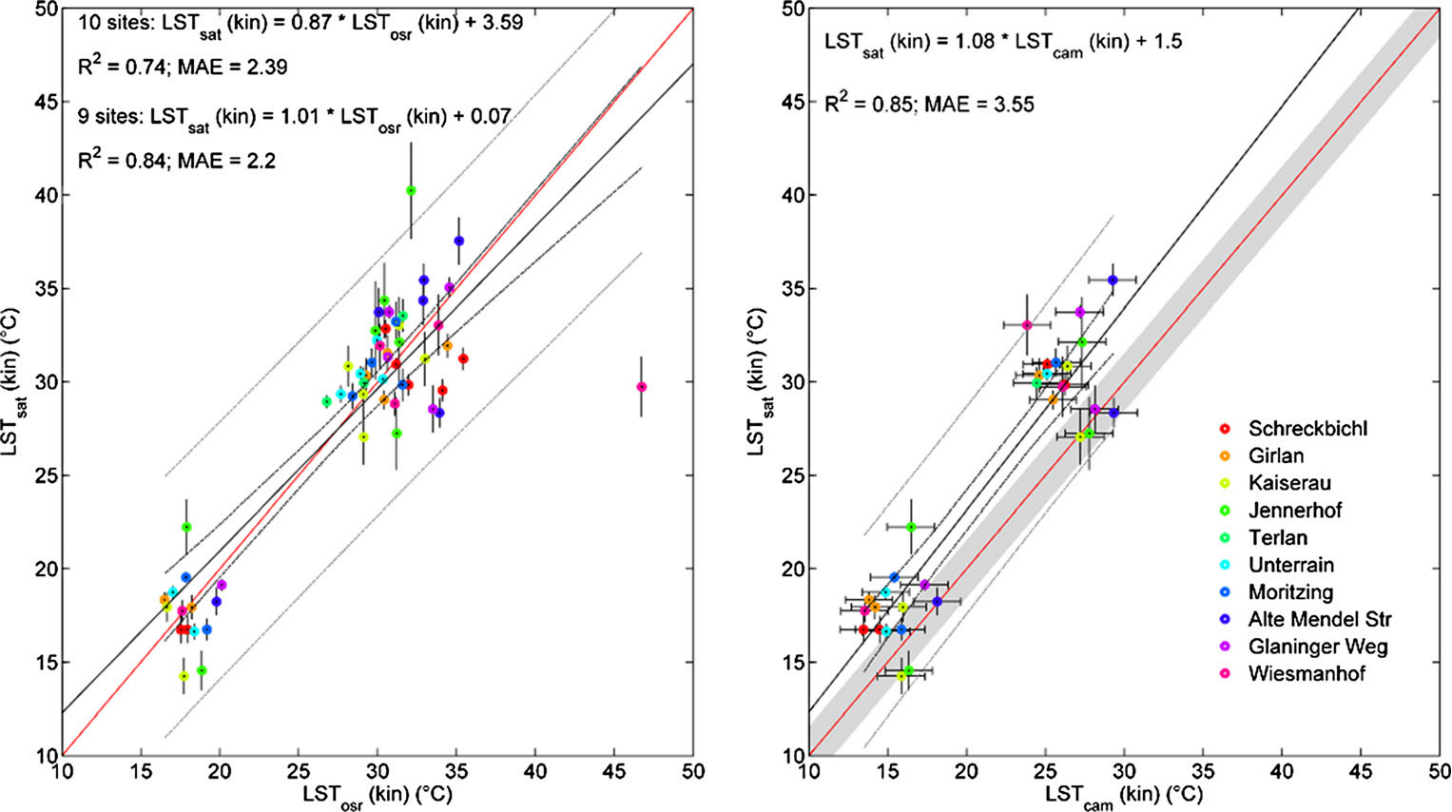
*Left panel*: intercomparison of kinetic land surface temperatures measured on site by radiometry (LST_osr_ (kin)) and from remote sensing (ASTER Level 2B03) (LST_sat_ (kin)). *Right panel*: intercomparison of kinetic land surface temperatures measured by ground-based TIR imagery (LST_cam_ (kin)) and from remote sensing (ASTER Level 2B03) (LST_sat_ (kin)). *Red line*: 1:1 line, *black solid line*: regression line, *black dashed line*: 95 % prediction interval of regression line, *grey dashed line*: 95 % prediction interval of observations, *grey shaded area*: ±1.5 K on 1:1 line marking camera accuracy. *Error bars* on LST_sat_ data refer to the standard deviation within a 3 × 3 pixel area centered around LST_osr_ locations. *Error bars* on LST_cam_ (kin) data refer to the camera accuracy of ±1.5 K

Generally, a good correlation of LST_osr_ (kin) and LST_sat_ (kin) could be found applying these transformations, whereas there is a distinct outlier datum in the Wiesmanhof dataset (Fig. 2, left panel). In contrast, comparing LST_cam_ (kin) with LST_sat_ (kin) did not reveal any outliers for the Wiesmanhof data (Fig. 2, right panel). But, as evident from Fig. 2, LST_cam_ (kin) and LST_sat_ (kin) estimates are clearly offset and show a higher mean absolute error (MAE) compared to LST_osr_ (kin) data.

**Fig. 3:**
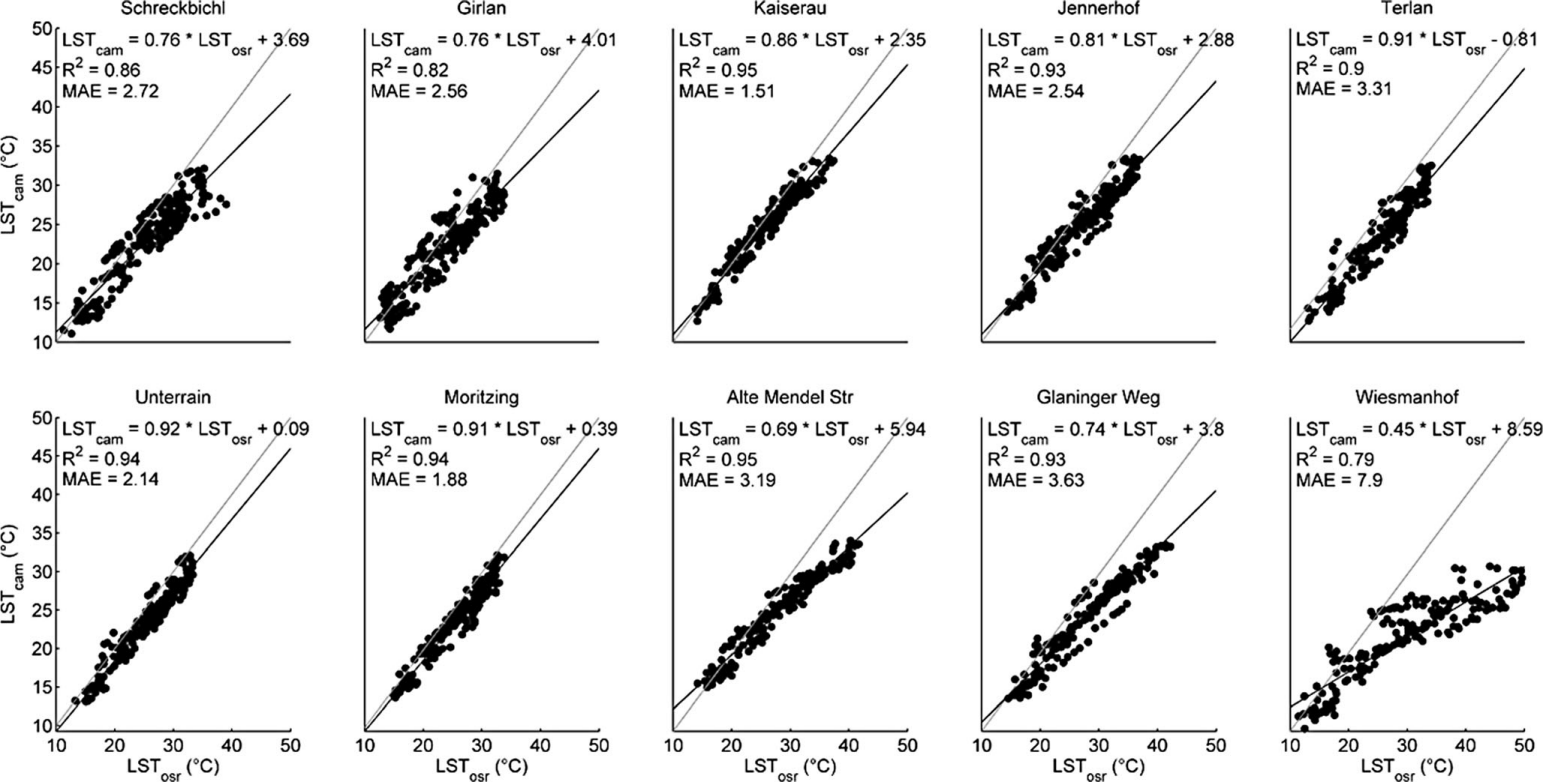
Correlations of land surface temperatures measured by on-site radiometery (LST_osr_) and ground-based TIR imagery (LST_cam_) per site including correlation statistics. *Grey dotted lines*: 1:1 line; *black bold lines*: sls-regression line

The Wiesmanhof site was excluded in any further analysis because of the following: (i) LST_osr_ (kin) does not always coincide with LST_sat_ (kin) at the Wiesmanhof site, while this site does not stand out when comparing LST_sat_ (kin) with LST_cam_ (kin) data; (ii) in nine out of ten cases LST_osr_ (kin) and LST_cam_ (kin) data are well correlated (except for the Wiesmanhof site at high temperatures) (Fig. 3); and (iii) photographs of the measured plot at Wiesmanhof (taken regularly at times of data collection or maintenance work; not shown) showed withered vegetation right below the sensor during periods with high air temperatures (end of July and around the 20th of August) while no dryness was observed at the rest of the meadow (plot not representative).

### LST_cam_ vs. LST_osr_

Radiant LST_cam_ and LST_osr_ were well correlated at nine out of our ten sites (Fig. 3). As mentioned in the previous paragraph, the Wiesmanhof field site was excluded from any further analyses. Slope and offset of the regression lines ranged from 0.69 to 0.92 and −0.81 to 5.94 K, respectively (Fig. 3). The coefficient of determination (*R* ^2^) and the MAE ranged from 0.82 to 0.95 and 1.51 to 3.63 K, respectively, with an average MAE of 2.61 K (Fig. 3).

As shown in Fig. 3, LST_cam_ are lower on average in all cases compared to LST_osr_, especially at higher temperatures, clearly indicating the necessity to account for atmospheric effects on LST measurements at landscape scales by TIR cameras.

**Fig. 4:**
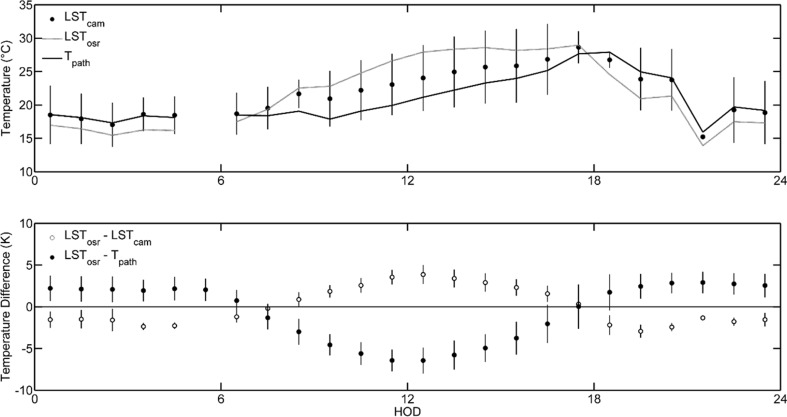
*Upper panel*: mean diel variations of land surface temperatures measured by on-site radiometry (LST_osr_) and by ground-based TIR imagery (LST_cam_), as well as path temperature (*T*_path_). Only data at times with LST_cam_ data available were used. *Lower panel*: mean diel variation of the differences between LST_osr_ and LST_cam_ as well as the differences between LST_osr_ and *T*_path_. *Error bars* refer to 1 stdv in any case. For reasons of clarity, *error bars* are shown for LST_cam_ data only in the *upper panel*

At all sites, uncorrected LST_cam_ is on average between 1.19 and 3.52 K lower than LST_osr_. While these average deviations appear to be rather small, the differences between LST_cam_ and LST_osr_ show a pronounced diel cycle. The observed differences between these two methods (Δ_LST_ = LST_osr_ − LST_cam_) ranged from −3.9 up to 11.5 K at the maximum. On average, Δ_LST_ was negative during the night time hours, ranging between −3 and −1 K. At sunrise, mean Δ_LST_ rose, reached its maximum of 3.9 K around noon, and decreased again from then on (Fig. 4).

**Fig. 5:**
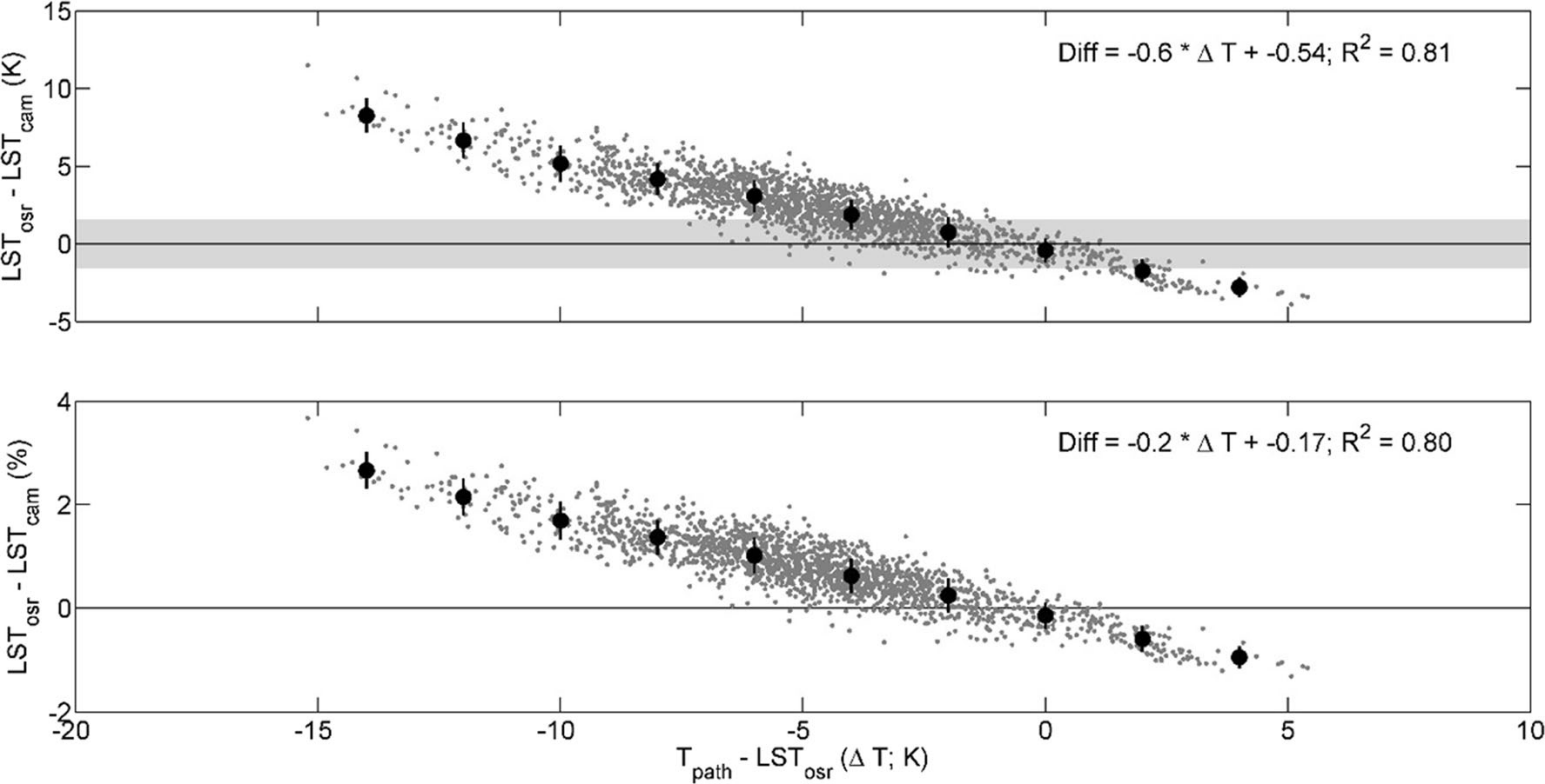
Correlations of the temperature difference between path temperature (*T*_path_) and land surface temperatures from on-site radiometry (LST_osr_) and the measurement difference between LST_osr_ and land surface temperatures from ground TIR imagery (LST_cam_) (Δ*T*) (*grey dots*). *Upper panel*: absolute difference; *grey horizontal bar* refers to ±1.5 K (camera accuracy). *Lower panel*: relative difference. *Big black dots* refer to bin averaged data including their error bar (1 stdv)

Given *T*_path_ from radiometer measurements, we calculated the difference between LST_osr_ and *T*_path_ (Δ*T*). Given Δ*T*, the residuals between LST_cam_ and LST_osr_ could be explained to a very large extent. Eighty-one percent of the residual variation is explained by Δ*T* (*n* = 1839; *p* < 0.01) (Fig. 5).

Correcting LST_cam_ data according to this correlation of the residuals with Δ*T* does result in slope and offset values ranging from 0.91 to 1.00 and −0.18 to 3.34 K, respectively. *R* ^2^ improved noticeably and ranged between 0.98 and 0.99, and the MAE was reduced from 2.61 K on average for uncorrected data to a range of 0.49 to 1.15 K (mean 0.74 K) for the nine sites.

While this finding does show the importance of atmospheric corrections on the data, this correlation is not relevant for any data correction as this method would require information on actual LST on landscape scale.

### LST model calibration and validation

In order to correct LST on landscape scale, a multiple linear regression model was set up to model LST_osr_ by the use of four independent variables (LST_cam_, LST_cam_ − *T*_path_, *T*_path_, and APL). Given a variance inflation factor (VIF) well above ten indicating multi-collinearity, *T*_path_ was excluded as an independent variable from further analysis. With VIFs lower than 1.33, none of the remaining three independent variables (LST_cam_, LST_cam_ − *T*_path_, and APL) gave evidence for further multi-collinearity (Kutner et al. 2003; Pan and Jackson 2008; Rogerson 2001). Furthermore, no scatterplot of dependent vs. independent variables revealed non-linear dependencies.

The three independent variables generated a highly significant model (*p* < 0.001) with a determination coefficient of 0.92 (adjusted *R* ^2^; root mean squared error (RMSE) = 1.7 K) based on ca. 50 % randomly chosen observations (calibration dataset). Residual analysis revealed no noticeable pattern (heteroscedasticity) and no obvious deviation from normal distribution.

Statistical validation of the model was done applying the model to the remaining 50 % of observation data, which resulted in an adjusted *R* ^2^ = 0.93 (LST_osr_ = 1.00 LST_osr predicted_ − 0.19; RMSE = 1.68 K).

**Fig. 6:**
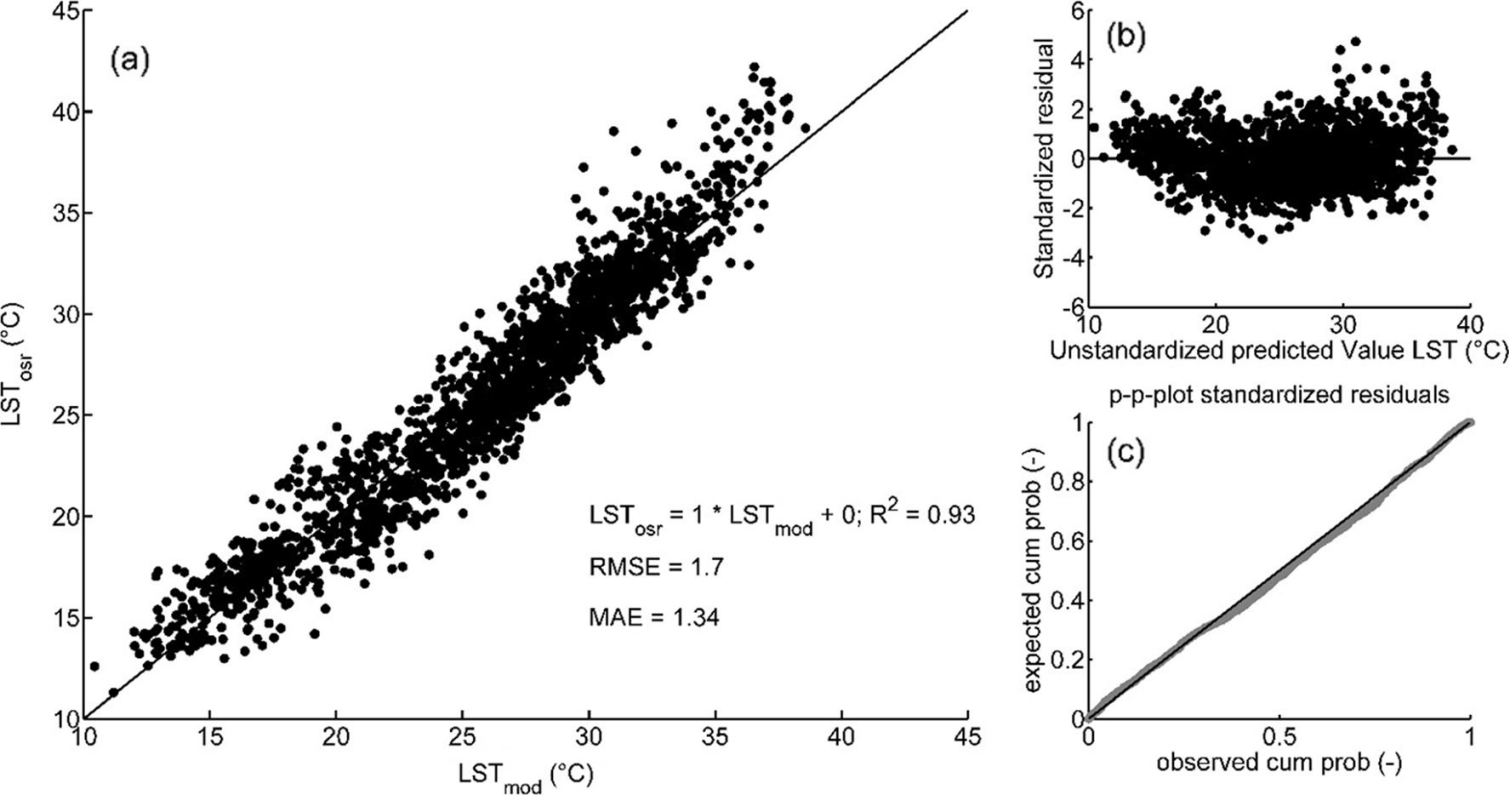
**a** Correlation of measured land surface temperatures from on-site radiometry (LST_osr_) with modelled LST including the regression line. **b** Standardized residuals vs. unstandardized predicted values. **c** p-p-plot of observed (*grey*) vs expected (*black*) cumulative residual distribution

Based on the available dataset (*n* = 1839) and the three selected independent variables LST_cam_, LST_cam_ − T_path_, and APL, the LST model was given by:

1 $$ {LST}_{osr\kern0.5em \mathrm{predicted}}=-3.971+1.086\kern0.5em {LST}_{cam}+0.767\left({LST}_{cam}-{T}_{\mathrm{path}}\right)+0.000469\kern0.5em APL, $$

representing a highly significant model for LST (*p* < 0.001; adj. *R* ^2^ = 0.93; RMSE = 1.70 K) (Fig. 6).

According to the standardized coefficients beta ($\overset{\sim }{\beta }$), LST_cam_ exerted the highest influence on the LST model, followed by the difference of LST_cam_ and *T*_path_ (LST_cam_ − *T*_path_) and atmospheric path length (APL) (Table 3).

**Table 3:** Three variables exhibited significance and were used in our final LST model

LST model	Unstandardized coefficients		Standardized coefficients $\tilde{\beta}$	*T*-value (t)	Significance (*p* value, two sided)	VIF
	*β*_*i*_	S.E.				
(Constant)	−3971	0.247		−16,060	0.000	
LST_cam_	1.086	0.008	0.909	142.350	0.000	1.086
LST_cam_ − *T*_path_	0.767	0.029	0.194	26.258	0.000	1.256
APL	0.000469	0.000	0.128	16.697	0.000	1.105

*S.E.* standard error, *VIF* variance inflation factor, *LST*_*cam*_ land surface temperatures measured by ground-based TIR imagery, *T*_*path*_ measurement path temperature, *APL* atmopsheric path length

### LST model application

Average differences of LST_osr_ and *T*_path_ during all measurement campaigns ranged from −6 to 10 K. To demonstrate consequences of these temperature differences, two different situations for one field of view were selected, including the stations Kaiserau, Jennerhof, Terlan, and Unterrain (scene 2). On 2 August 2012 at 11:30 CET, a mean difference between LST_osr_ of these sites and *T*_path_ of 6.7 K was observed (example 1), while on 24 August at 03:00 CET, these two temperatures differed by −2.5 K on average (example 2). These values represent rather high and low measured differences for that scene.

Presented in Table 4 are the meteorological conditions for the times of examples 1 and 2. Data presented in Table 4 represent average conditions for these specific dates of the year and times of the day. 2 August (example 1) was characterized by bright sunshine until the time of presented measurements, while on 24 August (example 2), it was partly cloudy around midday and clear sky conditions for the rest of the day. While LST_osr_ and T_air_ were relatively similar at the time of example 1, *T*_path_ was several degrees cooler on average, with differences ranging from −6 down to −11 K (Table 4). In contrast, at the time of example 2, the average *T*_path_ was 2.1 K warmer than the average LST_osr_, with differences ranging from −0.2 up to 3.5 K.

**Table 4:** Meteorological conditions on reference days 2 August 2012 11:30 (example 1; E1) and 24 August 2012 03:00 (example 2; E2)

Site		LST_osr_ (°C)		*T*_air_ 2 m (°C)		*T*_path_ (°C)		LST_cam_ (°C)		SR (W m^−2^)		Wind speed (m s^−1^)		RH (%)	
Nr.	Name	E1	E2	E1	E2	E1	E2	E1	E2	E1	E2	E1	E2	E1	E2
1	Schreckbichl	31.8	18.5	27.5	20.1	22.9	20.6	26.0	–	728	0	0.28	1.64	61	76
2	Girlan	30.1	17.7	28.4	20.0	23.1	20.7	25.5	20.6	–	0	1.24	0.68	57	77
*3*	*Unterrain*	*29.3*	*19.4*	*32.1*	*20.7*	*23.5*	*20.9*	*26.5*	*21.0*	*728*	*0*	*1.00*	*0.52*	*60*	*80*
*4*	*Terlan*	*29.9*	*18.1*	*29.7*	*19.6*	*23.5*	*20.9*	*25.7*	*20.6*	*684*	*0*	*0.28*	*0.52*	*55*	*84*
*5*	*Kaiserau*	*29.6*	*17.4*	*30.7*	*18.2*	*23.5*	*20.9*	*27.6*	*20.5*	*756*	*0*	*0.84*	*0.28*	*53*	*95*
*6*	*Jennerhof*	*32.2*	*18.7*	*30.7*	*20.1*	*23.5*	*20.9*	*28.6*	*20.8*	*764*	*0*	*0.84*	*0.84*	*48*	*82*
7	Moritzing	29.6	18.7	29.3	20.0	23.5	20.9	26.8	–	810	0	1.08	0.68	61	86
8	Alte Mendl Str	34.7	21.1	31.2	22.6	23.5	20.9	30.1	–	686	0	1.16	1.00	48	70
9	Glaninger Weg	32.6	18.7	28.3	20.7	23.0	20.6	28.7	–	828	0	1.48	0.76	49	75
	*Mean*	*31.1*	*18.7*	*29.8*	*20.2*	*23.4*	*20.8*	*27.3*	*20.7*	*762*	*0*	*0.91*	*0.77*	*55*	*81*

**Fig. 7:**
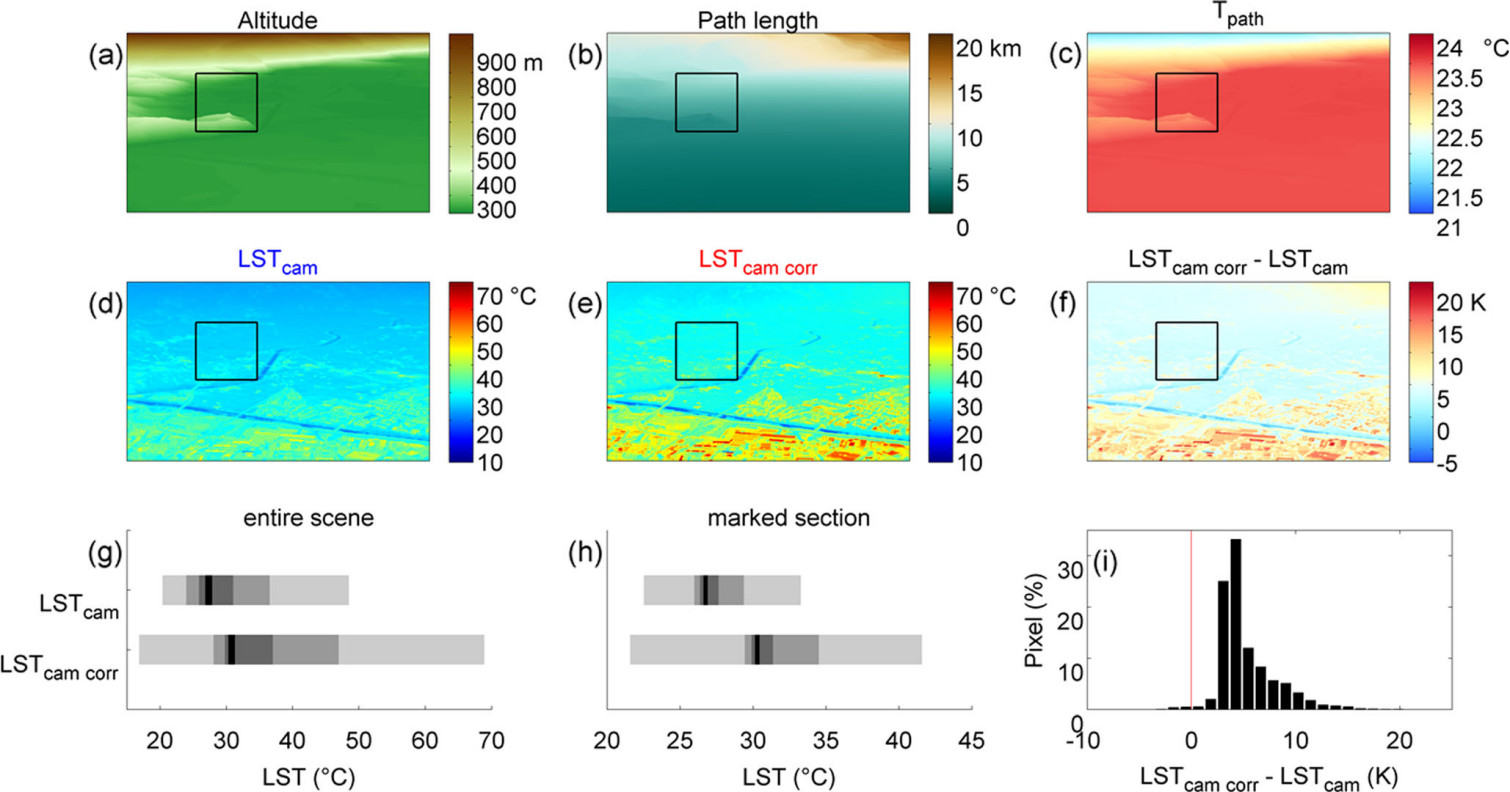
Example 1 (2 August 2012, 11:30 CET)—**a** elevation model, as seen by ground-based TIR imagery. **b** Resulting atmospheric path lengths (APL) for each pixel. **c** Average path temperatures (*T*_path_) for the time the infrared images were taken. **d** Land surface temperatures as measured by ground-based TIR imagery (LST_cam_). **e** Resulting LST_cam_ from model application (LST_cam corr_). **f** Difference between LST_cam_ and LST_cam corr_. **g** Temperature ranges of LST_cam_ and LST_cam corr_ for the entire scene. **h** Temperature ranges of LST_cam_ and LST_cam corr_ for the marked section in panels **a**–**f**. *Grey shadings* in **g** and **h** refer to min–max range, 90 % percentile, 50 % percentile (IQR), and the median (*black line*), respectively. **i** Histogram of the differences in panel **f** for the entire scene

**Fig. 8:**
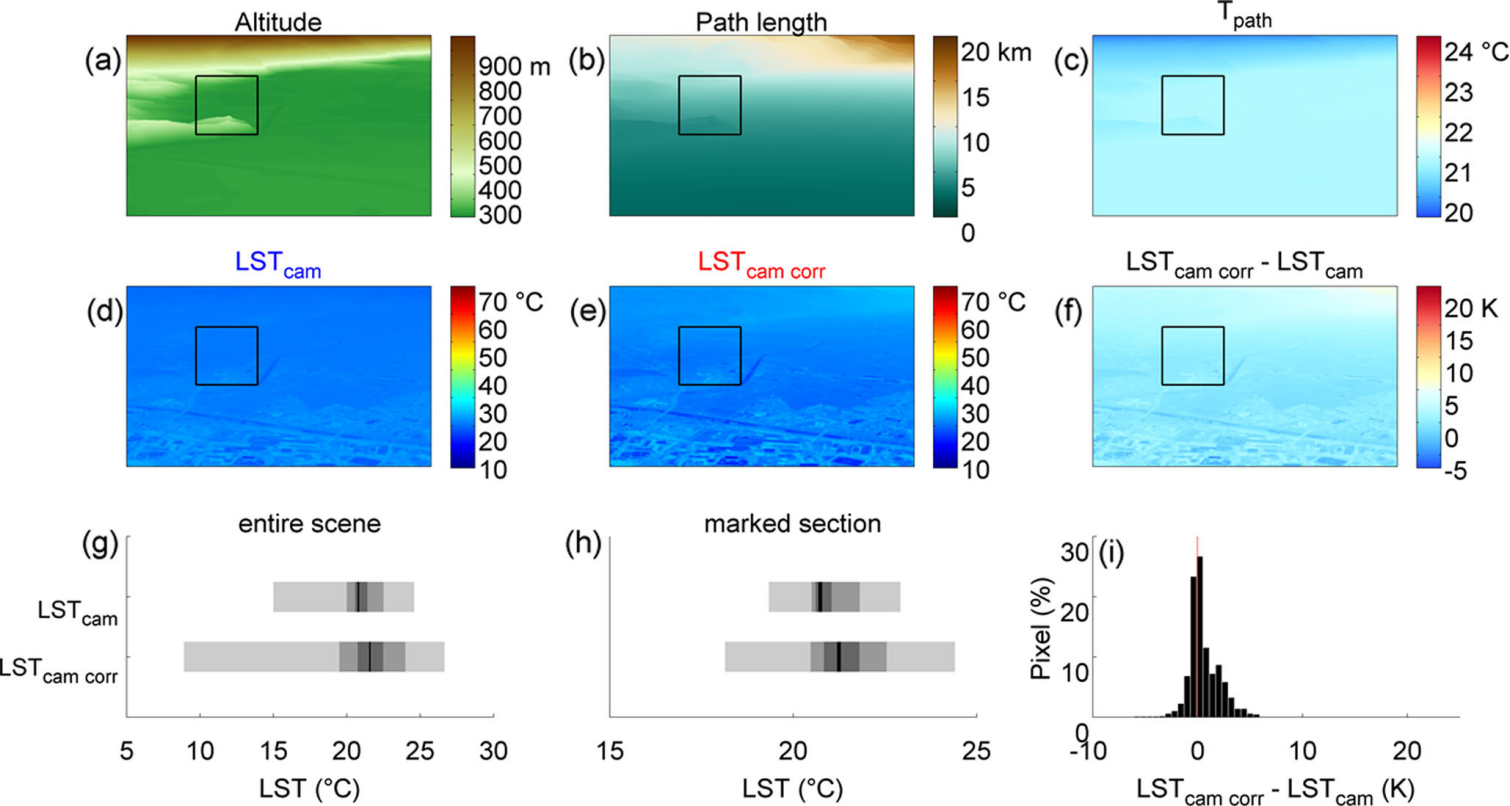
Example 2 (24 August 2012, 03:00 CET)—**a** elevation model, as seen by ground-based TIR imagery. **b** Resulting atmospheric path lengths (APL) for each pixel. **c** Average path temperatures (*T*_path_) for the time the infrared thermograms were taken. **d** Land surface temperatures as measured by ground-based TIR imagery (LST_cam_). **e** Resulting LST_cam_ from model application (LST_cam corr_). **f** Difference between LST_cam_ and LST_cam corr_. **g** Temperature ranges of LST_cam_ and LST_cam corr_ for the entire scene. **h** Temperature ranges of LST_cam_ and LST_cam corr_ for the marked section in panels **a**–**f**. *Grey shadings* in **g** and **h** refer to min–max range, 90 % percentile, 50 % percentile (IQR), and the median (*black line*), respectively. **i** Histogram of the differences in panel **f** for the entire scene

Italicized sites are covered by scene 2 shown in Fig. 7 and Fig. 8. Numbers (nr.) refer to numbers in Fig. 1

Consequences of these conditions on LST_cam_ and according corrections on these data by the LST model at landscape scale are shown in Fig. 7 and Fig. 8. The marked section in panels a–f was used to restrict data to areas covered by vegetation, as the model setup was done using data from such areas only. Results covering settlement or industrial areas (right and lower thermal image area, respectively) could thus not be validated.

This application of the LST model on landscape scale clearly shows that correcting for atmospheric influences (i) amplifies the measured LST spectrum (for the pronounced case in Fig. 7, the LST range was extended by as much as 10 K for the marked section) and (ii) shifts median temperatures depending on the difference between *T*_path_ and surface temperature.

## Discussion

Various studies on LST have been conducted using ground-based TIR cameras on landscape scale. These instruments gained popularity in ecosystem research due to their high temporal and spatial resolution as well as their operational simplicity (Corsi 2010; Pron and Bissieux 2004). In this study, we determined the magnitude of atmospheric effects on ground-based radiant surface temperature by comparison of LST_cam_ with LST_osr_. Furthermore, we established a multiple linear regression model to correct LST_cam_ data and to show the effects of *T*_path_ and APL on LST_cam_ data.

While LST_osr_ and LST_sat_ data did show a good correlation, LST_cam_ (kin) data, not corrected for atmospheric effects, were clearly offset compared to LST_sat_ data (Fig. 2). Once corrected for atmospheric effects, using our multiple linear regression model, LST_sat_ and LST_cam_ (kin) agreed reasonably well, reducing the MAE from 3.55 to 2.45 K (data not shown). Beside atmospheric effects, the offset could to some degree also be a result of thermal anisotropy, i.e., the LST depends on the viewing direction of the sensor (Christen et al. 2012; Kimes 1980; Lagouarde et al. 2000; Lagouarde et al. 2004; Voogt and Oke 2003). Under cloudless conditions, the satellite observes predominantly sunlit surfaces because of the nadir view. The oblique view of the TIR camera includes more vertical surfaces which could be cooler due to shading.

Focusing on the LST_osr_ and LST_cam_ comparison, we found that LST_cam_ clearly deviate from LST_osr_. Δ_LST_, the difference between LST_osr_ and LST_cam_, showed a pronounced diel cycle, and Δ_LST_ was negatively correlated (*p* < 0.001) with the difference between *T*_path_ and LST_osr_, with *T*_path_ being derived independently from microwave radiometer measurements. These findings show a strong influence of atmospheric properties along the measuring path on the LST_cam_ measurements. Using a multiple linear regression model, we could show that the measured range of LST_cam_ data is amplified when accounting for *T*_path_ and mean LST are shifted either positively or negatively, depending on the temperature difference between LST_osr_ and *T*_path_.

Comparing results of LST_osr_ with LST_cam_ of course holds the problem of finding the exact pixel covering the LST_osr_ site and the fact that these two methods differ in their spatial resolution. To check the validity of our results, we performed a sensitivity analysis on our comparison of methods. To this end, we defined a pixel region for any LST_osr_ site within a thermal image with the pixel most likely covering the LST_osr_ site centered within a 5 times 5 pixel domain (center pixel surrounded by 24 pixels). Given these regions, we ran our analysis (i) choosing the pixel within these regions matching the LST_osr_ readings worst and (ii) choosing the one matching the LST_osr_ reading closest. Even between these two extreme scenarios, the mean difference in the RMSE comparing LST_osr_ with LST_cam_ was as low as 0.16 K (range 0.0–0.63 K) and differences in *R* ^2^ were below 0.01 in any case. This low sensitivity on the exact pixel location can be attributed to the land cover in that region, which shows low variability at small scales due to the intensive pomiculture. The site being most sensitive to pixel localization was “Alte Mendel Strasse,” which is located in close proximity to the settlement area of Bozen/Bolzano.

Our findings of a distinct influence of the atmosphere on LST derived from ground-based TIR imagery are in accordance with numerous studies that report on the these influences from satellite-based measurements down to ground-based measurements with APL of some hundred meters (Chandrasekhar 1960; Jacob et al. 2003; Meier et al. 2011; Norman et al. 1995; Voogt and Oke 2003). Meier et al. (2011) and Wawrzyniak et al. (2013), for example, report a magnitude of atmospheric effects during a diel cycle of up to 6.7 K in an urban environment and over natural environments, respectively, at path lengths lower than 800 m. These effects are particularly noticeable under the conditions of a high surface-to-path temperature difference, a pattern described, e.g., by Meier et al. (2011) as well. In the present study, this difference alone explained 81 % of the variation in LST_cam_-LST_osr_ difference (*p* < 0.01) (Fig. 5).

Huge differences in temperature between a vegetated surface and the air above it may occur at times with (i) a high energy input, especially due to high incoming shortwave radiation (Kahmen et al. 2011; Lambers et al. 1998; Martin et al. 1999; Wilson et al. 1987), (ii) a low transpirational cooling due to water limitation (Camoglu 2013; Fuchs 1990; Gates 1964; Jackson et al. 1981), and (iii) low atmosphere-vegetation coupling (Jones 1992; McNaughton and Jarvis 1983).

Due to the increase in solar radiation and a decrease in air temperature with increasing altitude and the low atmospheric coupling of short alpine vegetation (Goldberg and Bernhofer 2008; Jarvis and Mcnaughton 1986; Tappeiner and Cernusca 1996), mountain landscapes facilitate high surface to air temperature differences. Furthermore, mountain regions feature high spatial variability in slope, aspect, and altitude, which in turn does lead to high spatial differences in the solar energy input (Bertoldi et al. 2010; Garnier and Ohmura 1968; Isard 1983) and thus LST.

Given these conditions, any measurements of LST in mountain landscapes, not accounting for atmospheric effects, do not only result in inaccurate absolute LST data but also underestimate the LST variability, both in space (Fig. 7 and Fig. 8) and time, due to the diel cycle in LST-*T*_path_ difference. Under these circumstances, the findings of, e.g., Scherrer and Körner (2010) may very likely even have underestimated the described high LST variability within their investigated area.

One could argue that the atmospheric effects on LST_cam_ data are lower than the camera accuracies. However, in order to stay below a measurement error of 1.5 K (camera accuracy), the absolute differences between LST_osr_ and *T*_path_ (Δ*T*) would have had to be lower than 4.6 and 2.1 K on average for our minimum APL (3260 m; Kaiserau) and our maximum APL (9038 m; Terlan), respectively (data not shown). Given the results shown in Fig. 5, Δ*T* reaches values as high as 15 K, which is well above the thresholds described above. Put another way, extrapolation of our data implies that an APL of less than 1000 m would be required to stay within the ±1.5 K range given by the accuracy of conventional TIR cameras.

The applied TIR camera is sensitive within a certain range of the electromagnetic spectrum, (7.5–14 μm). Hence, the proposed correction method is comparable with single-channel methods that are applied to satellite sensors with a single TIR band, e.g., LANDSAT (e.g., Sobrino et al. 2004). A standard approach to obtain LST is to solve the radiative transfer equation in the TIR spectrum. The atmospheric parameters, i.e., atmospheric transmissivity, between the surface and the sensor as well as down-welling and up-welling atmospheric radiance can be calculated from vertical profiles of atmospheric temperature and water vapor and using radiative transfer codes like MODTRAN (Berk et al. 2005; Berk et al. 1998). Our method has the advantage that we do not need the radiative transfer simulations, but due to the oblique view of the TIR camera at the landscape scale, we need to consider spatial variability of LOS parameters in contrast to a near nadir view of most satellite-based sensors. All single channel approaches need accurate data on temperature and water vapor distribution in the atmosphere between the surface and the sensor. Therefore, and as our study shows, on-site data about the vertical distribution of at least atmospheric temperature and a DEM of the study region should be available for LST studies on landscape scale by means of ground-based TIR imagery. This should be considered in the conception of experimental setups and field campaigns.

In a further study, we will compare our results with atmospheric correction methods based on simulations with MODTRAN and other models.

## Conclusions

While atmospheric corrections on LST measurements are routinely applied on remote sensing or airborne systems, such corrections had been neglected in many landscape-scale studies using TIR cameras, despite atmospheric measurement path lengths of several hundred or thousand meters. Based on our intensive field measurements and on our modelling results, we were able to show that neglecting the atmospheric effect on ground-based TIR imagery does lead to substantial measurement errors. We could demonstrate that, depending on the temperature difference between the land surface and the overlying air masses, these errors are relevant even at relatively short measurement paths and particularly for spatially varying LOS parameters due to an oblique view of the TIR camera. Furthermore, our results suggest that differences in LST on landscape scale are underestimated in both spatial and temporal domains, due to the dampening effect of the atmosphere on the LST measurements.
